# Age-related variations in trunk muscle activation and kinematics during lifting in chronic low back pain: a cross-sectional study

**DOI:** 10.1038/s41598-025-04780-0

**Published:** 2025-06-23

**Authors:** Tianwei Zhang, Ali Firouzabadi, Daishui Yang, Sihai Liu, Lukas Mödl, Hendrik Schmidt

**Affiliations:** 1 Julius Wolff Institute, Berlin Institute of Health at Charité - Universitätsmedizin Berlin, Augustenburger Platz 1, 13353 Berlin, Germany; 2 Institute of Biometry and Clinical Epidemiology, Charité - Universitätsmedizin Berlin, Charitéplatz 1, 10117 Berlin, Germany; 3 Department of Orthopaedic Surgery, Sir Run Run Shaw Hospital, Zhejiang University School of Medicine, Hangzhou, P. R. China

**Keywords:** Electromyography, Manual material handling, Functional assessment, Motion analysis, Rehabilitation, Aging, Chronic pain, Skeletal muscle, Orthopaedics, Geriatrics

## Abstract

Chronic low back pain (cLBP) is a leading cause of disability worldwide, however, the influence of age on electromyography (EMG) during lifting tasks is not well understood. This study examined the effects of age and pain on EMG and kinematics in 102 participants. They were divided into no low back pain (no-BP) (*n* = 42; mean age: 41.86) and cLBP groups (*n* = 60; mean age: 43.41) and further categorized by age: 44 under 40 years (mean age: 31.14) and 58 over 40 years (mean age: 51.38). Two lifting tasks from the ground to the hip height were performed: lifting a 10 kg box in front of the body (Task 1) and two 5 kg dumbbells beside the body (Task 2), with EMG and flexion angles recorded. Older participants showed significantly higher EMG amplitudes (*p* < 0.05), particularly in Task 1 while holding the weight at hip height. No significant EMG differences were found between cLBP and no-BP groups after adjusting for age, sex, and body mass index (BMI) (*p* > 0.05). Task 1 showed higher back muscle activation than Task 2 (*p* < 0.05). These findings suggest that age, rather than pain, may play a more critical role in muscle activation, highlighting the need for age-specific interventions in cLBP rehabilitation.

## Introduction

 Chronic Low Back Pain (cLBP) is a major contributor to global disability, substantially affecting individuals’ quality of life and imposing significant economic burdens due to healthcare expenditures and lost productivity from work absenteeism^1,2^. Manual material handling, such as lifting and carrying, has been identified as a key physical risk factor for the development of cLBP^3–6^. These tasks increase the mechanical stress and strain placed on the spine, raising the risk of injury^6,7^. Studies have shown that lifting weights beside the body, rather than in front, considerably reduces low back loading, which may help in preventing both injury and re-injury to the lumbar region^8–10^. For instance, Subramaniyam et al.^8^ demonstrated that lifting loads beside the body significantly decreases back muscle activity by as much as 38–91%, highlighting the importance of load positioning in minimizing muscular strain during lifting tasks. This reduction in muscular effort is particularly beneficial in lowering the burden on trunk muscles. However, while these findings offer valuable insights into back muscle mechanics in individuals without low back pain (no-BP), the electromyographic (EMG) activity of abdominal muscles and the specific effects on cLBP patients remain insufficiently explored. Understanding these factors is crucial for developing more effective rehabilitation protocols for cLBP patients.

Lifting tasks are widely utilized in musculoskeletal assessments to evaluate functional capacity, especially in individuals with cLBP, as these tasks closely simulate daily activities that frequently cause or worsen pain^7,10–18^. Numerous studies have investigated differences in trunk muscle activations during lifting tasks, with a focus on muscle activation timing and EMG amplitude^19–22^. Several studies have shown that cLBP individuals demonstrate delayed muscle activation compared to no-BP group^19,23^. In contrast, Ferguson et al.^18^ found that the cLBP group demonstrated significantly earlier bilateral activation of the erector spinae (ES) muscles, with no differences in peak activation between groups. Similarly, Marras et al.^17,24^ observed increased activity in all back muscles in cLBP patients, while Courbalay et al.^25^ found higher ES activation during the lifting and lowering of heavy weights (20 lbs), though no significant differences in kinematics were found between the groups. Larivière et al.^16^ further reported that cLBP individuals exhibited reduced lumbar erector spinae (ESL) activity during the lowering phase and increased thoracic erector spinae (EST) activity during both lifting and lowering phases, again with no notable kinematic differences between groups. These findings suggest that variations in muscle recruitment patterns, rather than movement mechanics, may play a critical role in the pain and functional limitations observed in cLBP patients.

Most studies have predominantly focused on younger populations, often overlooking the impact of age on EMG patterns. Age-related musculoskeletal changes, including sarcopenia, reduced muscle strength, diminished flexibility, and decreased endurance, significantly influence functional capacity in older adults^26^. These changes can alter muscle recruitment strategies during physical tasks such as lifting. Additionally, as individuals age, the occurrence of cLBP becomes more common, with significant reductions in lumbar flexibility, particularly in flexion and extension, typically beginning around the age of 40 ^27,28^. This highlights the importance of considering age as a crucial factor in EMG analyses and musculoskeletal assessments.

However, current research lacks a thorough understanding of how age affects trunk muscle activity and kinematics during lifting tasks in individuals with cLBP. This study aims to address this gap by examining the effects of age and pain on EMG and kinematic patterns, while also exploring how lifting loads beside the body versus in front of the body impacts these variables. We hypothesize that: (1) individuals with cLBP will exhibit significant alterations in EMG patterns and trunk flexion angles compared to those without cLBP; (2) age will significantly modulate these EMG and kinematic changes; and (3) different lifting tasks will yield distinct EMG activation patterns.

## Method

### Study participants and ethics approval

This study was conducted at the Julius Wolff Institute, Berlin Institute of Health at Charité – Universitätsmedizin Berlin, Germany, from January 2022 to June 2024 as part of a four-year prospective project investigating factors affecting the development of cLBP. Ethical approval was obtained from the Ethics Committee of Charité – Universitätsmedizin Berlin (registry numbers: EA4/011/10, EA1/162/13), and the study was prospectively registered (DRKS-ID: DRKS00027907). All methods were performed in accordance with the Declaration of Helsinki.

All participants gave written informed consent after being fully briefed on the study procedures, and informed consent was also obtained for the online open-access publication of identifiable images. The inclusion criteria required individuals aged 19 to 64 years with a body mass index (BMI) under 29 kg/m². We conducted our study based on previous research that considered 40 years as a threshold for age sub-grouping^29,30^. Participants in the no-BP group had no history of back or pelvic pain and had not undergone any surgery on the spine or lower limbs. The cLBP group included patients with chronic low back pain lasting more than 3 months, with pain levels measured using the Numeric Rating Scale (NRS) ranging from 0 (no pain) to 10 (worst pain). Exclusion criteria for the cLBP group included a history of vertebral fractures, radiculopathy with muscle weakness, prior spinal surgery, and other conditions that limit daily activity, such as chronic obstructive pulmonary disease (COPD), heart failure, neurological disorders, or cancer^30^.

### Measurement devices and instrumentation

A Vicon Motion Capture System (Vicon Motion Systems, Inc., Oxford, UK) was utilized to capture 3D motion at a sampling frequency of 200 Hz. Twelve infrared cameras recorded the movement of 41 retro-reflective markers (14 mm in diameter) placed on key anatomical points, following the Vicon plug-in gait full-body marker protocol^31^.

Additionally, muscle activity was measured using a wireless EMG system (Myon Aktos, Schwarzenberg, Switzerland) with a sampling rate of 1000 Hz. Prior to placing the electrodes, the skin was shaved, cleaned, and disinfected with alcohol. EMG data were integrated into the Vicon Nexus system and synchronized with the Vicon data. Twelve surface EMG sensors were used to record muscle activity from the left and right multifidus (L/RMF) (~ 2 cm lateral to the midline at L5), left and right lumbar erector spinae (L/RESL) (~ 3 cm lateral to the midline at L3), and left and right thoracic erector spinae (L/REST) (~ 5 cm lateral to the midline at T9)^30^left and right external obliques (L/REO) (~ 10 cm lateral to the midline above the umbilicus, aligned with the muscle fibers), left and right internal obliques (L/RIO) (below the external oblique sensors, just above the inguinal ligament), and left and right rectus abdominis (L/RRA) (~ 3 cm lateral to the midline above the umbilicus)^7,10^. To reduce noise and artifacts, a band-pass filter (30–450 Hz) with a fourth-order Butterworth design was applied, and a notch filter was used to eliminate 50 Hz interference. After filtering, the EMG signals were full wave rectified, and the root-mean-square (RMS) envelopes were calculated using a 150-ms moving window. The RMS values were then normalized to the peak values from the maximal voluntary contraction (MVC)^7,30^.

### Study protocol

Participants underwent a clinical examination by an experienced orthopedic and trauma specialist and completed the following questionnaires: the Roland-Morris Disability Questionnaire (RMDQ)^32^ to evaluate the disability, the Tampa Scale for Kinesiophobia (TSK)^33^and the Fear-Avoidance Beliefs Questionnaire (FABQ)^34^ to assess fear of movement and avoidance beliefs (Table 1).

**Table 1:** Demographics of study participants.

	Age<40 (*n* = 44; 20 no-BP, 24 cLBP)	Age>40 (*n* = 58; 22 no-BP, 36 cLBP)	*P*-value	no-BP (*n* = 42; 20 Age<40, 22 Age>40)	cLBP (*n* = 60; 24 Age<40, 36 Age>40)	*P*-value
Sex(F/M)	20/24	31/27		22/20	29/31	
Years	31.14 ± 4.99	51.38 ± 7.31	**< 0.01**	41.86 ± 13.23	43.41 ± 10.97	0.52
Height (m)	1.76 ± 0.10	1.74 ± 0.10	0.30	1.73 ± 0.09	1.76 ± 0.10	0.61
Weight (kg)	71.67 ± 12.39	70.87 ± 10.66	0.73	70.52 ± 11.00	71.70 ± 11.71	0.18
BMI (kg/m²)	23.04 ± 2.70	23.34 ± 1.97	0.52	23.43 ± 2.39	23.06 ± 2.26	0.42
Pain Intensity	0.91 ± 1.70	1.53 ± 1.93	0.13	-	2.15 ± 2.04	-
RMDQ (0–24)	3.20 ± 3.90	4.45 ± 4.31	0.14	-	6.17 ± 3.85	**-**
TSK (11–44)	13.23 ± 11.52	14.21 ± 10.00	0.65	-	20.92 ± 5.70	**-**
FABQ-PA (scale 0–24)	9.50 ± 5.44	8.38 ± 6.01	0.43	-	9.73 ± 5.61	**-**
FABQ-w (scale 0–42)	12.46 ± 8.90	8.05 ± 6.95	**0.03**	-	10.32 ± 7.74	-
Task Time 1	3.92 ± 1.63	4.20 ± 1.12	0.89	3.64 ± 1.29	4.39 ± 1.34	**0.01**
Task Time 2	4.07 ± 1.47	3.86 ± 1.44	0.32	3.54 ± 1.42	4.25 ± 1.41	**0.01**

Abbreviations: BMI: Body Mass Index, RMDQ: Roland-Morris Disability Questionnaire, FABQ-PA: Fear-Avoidance Beliefs Questionnaire - Physical Activity Subscale, FABQ-w: Fear-Avoidance Beliefs Questionnaire - Work subscale, Task time 1: The time from standing to lifting the box to hip height in Task 1, Task time 2: The time from standing to lifting the box to hip height in Task 2, cLBP: chronic low back pain, no-BP: no chronic low back pain. Significant differences (*p* < 0.05) are shown in bold.

All participants performed a series of MVC tests and lifting tasks. For the MVC assessments of the three back muscles, subjects were positioned prone with their upper body extending over the edge of the table, while their legs remained straight and securely fixed. Participants were instructed to raise their head, shoulders, and elbows off the table, while the examiner, positioned at the subject’s head, applied symmetrical manual resistance to the shoulders, prompting maximal exertion^35^. For the rectus abdominis (RA) and internal oblique (IO) muscles, participants lay supine with their knees flexed at 90 degrees and hands placed on their chest. They performed a resisted curl-up, during which the experimenter, standing at the head of the bed, applied maximal isometric resistance symmetrically through the shoulders^35^. For external oblique (EO) evaluation, subjects lay on their side with legs straight and fixed and hands on the chest. They performed a resisted lateral curl-up with the investigator applying resistance through the shoulder^36^.

**Fig. 1:**
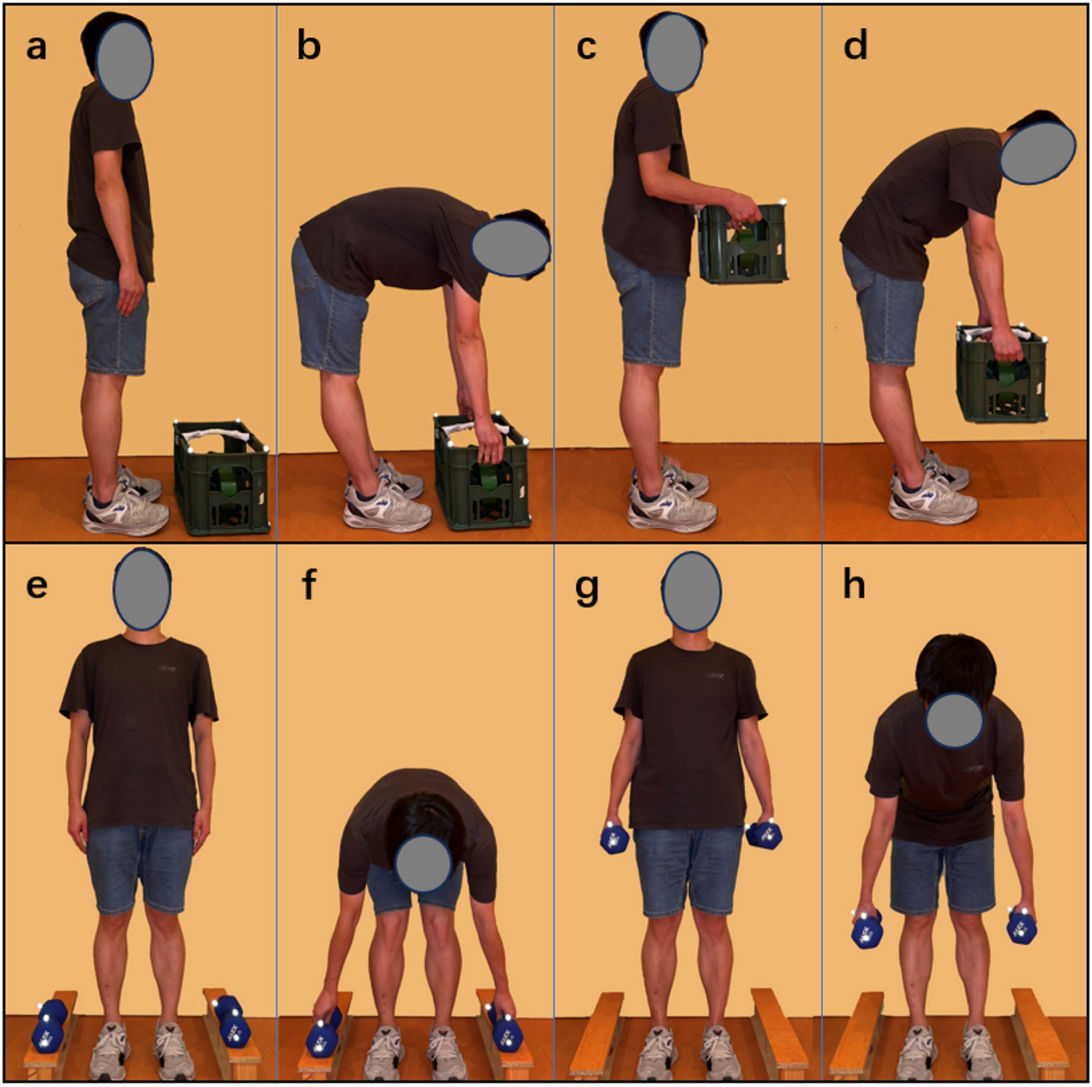
Lifting a 10 kg box in front of the body to hip height (Task 1, **a**-**d**), the lifting phase (**b**), the holding phase (**c**), and the lowering phase (**d**). Lifting two 5 kg dumbbells beside the body to hip height (Task 2, **e**-**f**), the lifting phase (**f**), the holding phase (**g**), and the lowering phase (**h**).

Participants took part in a practice session before the trials to ensure smoother movement during the tasks. Then, the participants were instructed to perform two symmetric lifting tasks from floor to hip height in the sagittal plane (Fig. 1): lifting a 10 kg box in front of the body to hip height (Task 1) and lifting two 5 kg dumbbells beside the body to hip height (Task 2)^7^. All tasks were initiated from a relaxed standing position, with the feet al.igned at shoulder width and the knees straight. From this starting position, participants bent forward, lifted the weight with self-selected velocity, returned to the upright standing position, held this position for 3 s, and then placed the box back down on the floor, returning to the initial position. Each task was repeated three times, with a one-minute rest period separating the trials^7,10^.

### Data processing

For kinematic analysis, flexion angles of the lumbar, thoracic, and pelvic regions at maximum trunk inclination (MaxP/L/T) were computed. The Vicon Nexus 2.8.1 system with the plug-in gait model was utilized to identify relevant frames and calculate the segmental angles. The lumbar flexion angle was determined by the intersection of the sagittal thoracic and pelvic axes, using the fixed transverse axis of the pelvis as the reference point. The thoracic flexion angle was defined as the angle between the projected sagittal thoracic axis and the sagittal laboratory axis. Similarly, the pelvic angle was defined as the angle between the projected sagittal pelvic axis and the sagittal laboratory axis^30,31^.

MATLAB R2020b (The MathWorks, Inc.) was used to process the EMG data. The onset times of twelve muscles were recorded. Muscle activation onset was visually identified by two trained examiners as the first noticeable rise in EMG activity above baseline that persisted for at least 50 milliseconds^37^. Our method of determining EMG onset involved normalizing the lifting cycle from an upright standing posture through lifting and placing down the weight and back to the upright standing posture. Thereby accounting for variations in movement velocity and ensuring consistency across participants. Differences in muscle activation timing were compared using the percentage of the onset point relative to the entire movement. Additionally, the EMG amplitude was measured during three phases: (1) lifting phase: lifting the weight to hip height, (2) holding phase: holding the weight at hip height, and (3) lowering phase: lowering the weight back to the floor (Fig. 1).

### Statistical analysis

Independent samples T-tests were performed to compare demographic and clinical variables between groups, ensuring comparability and providing descriptive statistics. Multivariate analysis of covariance (ANCOVA) was employed to evaluate the effects of age and pain status on EMG activity and flexion angles, with sex^10,29^and BMI^38^ included as covariates to control for potential confounding effects. When evaluating the impact of age on EMG activity and flexion angles, we controlled the pain status, sex, and BMI as covariates. In assessing the effect of pain status, we included age, sex, and BMI as covariates. To examine the interaction between age and pain status, we controlled sex and BMI as covariates. We applied logarithmic transformations to the data to stabilize variances and approximate a normal distribution. We conducted an Analysis of Variance (ANOVA) to compare the EMG differences between the two tasks, separately for the age subgroups and the pain subgroups, respectively.

All statistical analyses were performed using SPSS software (version 20, SPSS Inc., Chicago, USA). The statistical significance level was set at *p* < 0.05, and effect sizes were reported where applicable to quantify the practical significance of the findings. Partial eta squared (η²) was reported to quantify the effect sizes for significant effects identified in the ANCOVA. An η² value of 0.01 reflects a small effect size, 0.06 represents a moderate effect, and 0.14 signifies a large effect^39^. The sample size for this study was determined using G*Power 3.1.9.4, employing an a priori power analysis for an ANCOVA (fixed effects, main effects, and interactions) model. The calculation was based on an expected effect size (f) of 0.3, a significance level (α) of 0.05, and a statistical power (1-β) of 0.80. The analysis yielded a required total sample size of 90, with a denominator degree of freedom of 86 and an actual power of 0.803.

## Result

**Fig. 2:**
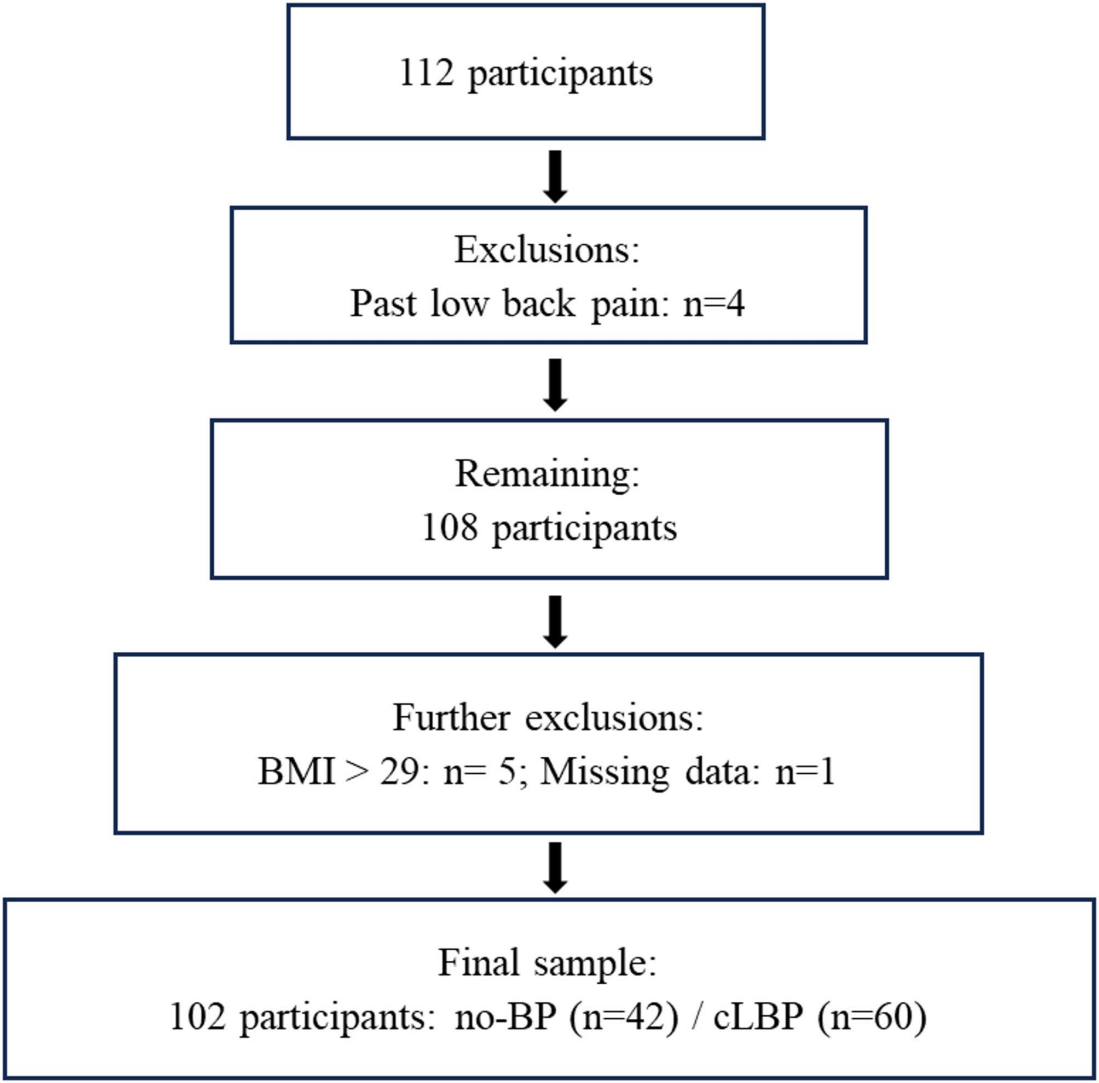
The flowchart of the participant selection and exclusion criteria. Abbreviations: no-BP: no low back pain, cLBP: chronic low back pain, BMI: body mass index. Past low back pain: Individuals with a history of low back pain are currently symptom-free.

A total of 112 participants were measured, with four exclusions due to past cLBP. Of the 108 eligible participants, further exclusions were made for BMI > 29 and missing EMG data, resulting in a final sample of 102 participants who successfully completed all assessments (Fig. 2). They were categorized into no-BP (22 f/20 m; mean age: 41.86) and cLBP groups (29f/31 m; mean age: 43.41). Additionally, participants were further divided by age: 44 participants were under 40 years old (mean age: 31.14), and 58 were over 40 (mean age: 51.38). Participant demographics are outlined in Table 1. No significant differences were observed in height, weight, and BMI between the groups (all *p* > 0.05). Furthermore, pain intensity did not differ significantly between participants aged under and over 40 years (*p* > 0.05). Notably, individuals over 40 exhibited a significantly lower FABQ-W score compared to their younger counterparts (*p* = 0.03). Additionally, significant differences in flexion velocity were observed between the cLBP and no-BP groups, with the cLBP group performing lifting tasks at a slower rate (*p* = 0.01 for Task 1; *p* = 0.01 for Task 2) (Table 1).

### Task1 results

#### EMG amplitude during the lifting phase

Significant EMG amplitude differences were found for LESL (*p* = 0.03, η² = 0.05) and LEO (*p* = 0.04, η² = 0.04) when assessing the effect of age, with pain controlled as a covariate. In contrast, no notable differences were observed when focusing solely on the effect of pain. However, considering the combined influence of both age and pain, significant differences were observed in RMF (*p* = 0.03, η² = 0.05) and LIO (*p* = 0.04, η² = 0.04) (Table 2).

**Table 2:** The EMG amplitude during the lifting phase in task 1.

EMG (MVC%)	<40	>40	no-BP	cLBP	Age		Pain		Age*Pain	
	Mean ± SD	Mean ± SD	Mean ± SD	Mean ± SD	η²	*P*	η²	*P*	η²	*P*
LMF	53.04 ± 15.84	59.37 ± 21.23	57.77 ± 23.62	55.78 ± 15.67	0.03	0.08	< 0.01	0.71	0.03	0.09
RMF	50.98 ± 19.9	57.02 ± 14.88	53.59 ± 19.37	54.91 ± 16.11	0.04	0.05	< 0.01	0.59	0.05	**0.03**
LESL	52.12 ± 22	60.47 ± 15.28	57.25 ± 19.95	56.51 ± 18.3	0.05	**0.03**	< 0.01	0.82	< 0.01	0.60
RESL	51.61 ± 21.36	58.11 ± 21.19	55.67 ± 26.24	54.98 ± 17.55	0.03	0.11	< 0.01	0.95	0.02	0.16
LEST	43.23 ± 24.37	54.1 ± 30.68	45.47 ± 25.02	52 ± 30.55	0.03	0.07	0.02	0.22	0.01	0.31
REST	47.33 ± 27.18	54.04 ± 31.74	47.26 ± 26	53.75 ± 32.22	0.01	0.28	0.02	0.19	0.03	0.11
LIO	9.19 ± 7.29	11.3 ± 14.54	10.85 ± 12.33	10.04 ± 11.71	0.02	0.21	< 0.01	0.85	0.04	**0.04**
RIO	8.36 ± 5.85	12.56 ± 18.67	8.92 ± 7.43	11.96 ± 17.93	0.02	0.21	0.01	0.33	< 0.01	0.91
LEO	7.16 ± 4.91	9.81 ± 7.63	8 ± 5.66	9.1 ± 7.31	0.04	**0.04**	0.01	0.46	0.01	0.48
REO	8.97 ± 9.17	10.82 ± 9.21	10.49 ± 9.86	9.67 ± 8.77	0.01	0.37	< 0.01	0.60	< 0.01	0.69
LRA	5.88 ± 4.08	7.66 ± 6.96	6.44 ± 6.02	7.18 ± 5.88	0.02	0.23	0.01	0.29	0.02	0.21
RRA	6.71 ± 9.13	7.13 ± 5.48	7.31 ± 9.65	6.69 ± 5.11	< 0.01	0.68	< 0.01	0.67	< 0.01	0.81

EMG: electromyography, MVC: maximal voluntary contraction, L/RMF: left/right multifidus, L/RESL: left/right lumbar erector spinae, L/REST: left/right thoracic erector spinae, L/REO: left/right external oblique, L/RIO: left/right internal oblique, and L/RRA: left/right rectus abdominis. η²: Partial eta squared. Significant differences (*p* < 0.05) are shown in bold. Covariates were adjusted as follows: for the effect of age, pain status, sex, and BMI were controlled; for the effect of pain status, age, sex, and BMI were controlled; and for the age-pain status interaction, sex and BMI were controlled.

#### EMG amplitude during the holding phase

For the EMG amplitude during the holding phase, when analyzing the effect of age and controlling the influence of pain, significant differences were observed in LMF (*p* < 0.01, η² = 0.10), RMF (*p* < 0.01, η² = 0.08), RESL (*p* = 0.02, η² = 0.06), LEST (*p* = 0.01, η² = 0.08), LEO(*p* = 0.04, η² = 0.04), and REO (*p* = 0.03, η² = 0.05). Conversely, when focusing solely on the effect of pain and excluding the influence of age, significant differences were observed only in RRA (*p* = 0.03, η² = 0.05). When considering the combined effects of age and pain, significant differences were observed only in the RMF (*p* = 0.04, η² = 0.04) (Table 3).

**Table 3:** The EMG amplitude during the holding phase in task 1.

EMG (MVC%)	<40	>40	no-BP	cLBP	Age		Pain		Age*Pain	
	Mean ± SD	Mean ± SD	Mean ± SD	Mean ± SD	η²	*P*	η²	*P*	η²	*P*
LMF	27 ± 10.07	34.26 ± 12.3	31.74 ± 14.11	30.61 ± 10.18	0.10	**< 0.01**	< 0.01	0.64	0.02	0.15
RMF	26.11 ± 11.04	31.68 ± 9.67	28.84 ± 11.83	29.51 ± 9.77	0.08	**< 0.01**	< 0.01	0.69	0.04	**0.04**
LESL	26.1 ± 17.6	30.75 ± 11.65	30.43 ± 18.4	27.52 ± 11.45	0.02	0.18	0.01	0.28	0.01	0.47
RESL	21.94 ± 11.45	27.71 ± 12.25	25.3 ± 13.91	25.1 ± 10.98	0.06	**0.02**	< 0.01	0.91	0.01	0.44
LEST	26.76 ± 16.03	33.81 ± 17.37	29.49 ± 15.83	31.57 ± 17.97	0.08	**0.01**	< 0.01	0.57	< 0.01	1.00
REST	27.36 ± 14.91	31.52 ± 18.72	30.59 ± 16.21	29.08 ± 17.95	0.02	0.22	< 0.01	0.78	0.02	0.15
LIO	8.46 ± 7.4	8.79 ± 11.71	7.74 ± 6.56	9.27 ± 11.83	< 0.01	0.72	0.01	0.37	0.03	0.11
RIO	7.2 ± 5.84	11.12 ± 16.49	7.41 ± 7.5	10.77 ± 15.69	0.02	0.19	0.02	0.21	< 0.01	0.73
LEO	5.33 ± 4.41	7.92 ± 7.43	5.67 ± 4.59	7.55 ± 7.32	0.04	**0.04**	0.02	0.16	0.01	0.43
REO	5.58 ± 3.92	8.83 ± 8.68	6.82 ± 5.12	7.8 ± 8.3	0.05	**0.03**	< 0.01	0.54	< 0.01	0.53
LRA	3.37 ± 3.27	4.49 ± 3.98	4.1 ± 4.57	3.93 ± 3.03	0.04	0.05	0.02	0.19	0.02	0.16
RRA	3.16 ± 2.47	4.58 ± 4.6	3.71 ± 4.63	4.13 ± 3.26	0.02	0.14	0.05	**0.03**	0.02	0.21

EMG: electromyography, MVC: maximal voluntary contraction, L/RMF: left/right multifidus, L/RESL: left/right lumbar erector spinae, L/REST: left/right thoracic erector spinae, L/REO: left/right external oblique, L/RIO: left/right internal oblique, and L/RRA: left/right rectus abdominis. η²: Partial eta squared. Significant differences (*p* < 0.05) are shown in bold. Covariates were adjusted as follows: for the effect of age, pain status, sex, and BMI were controlled; for the effect of pain status, age, sex, and BMI were controlled; and for the age-pain status interaction, sex and BMI were controlled.

#### EMG amplitude during the lowering phase

During the lowering phase, significant differences were observed only in RMF (*p* = 0.02, η² = 0.05) when isolating age while controlling pain. Conversely, neither pain alone nor the combined effects of age and pain resulted in significant differences (Table 4).

**Table 4:** The EMG amplitude during the Lowering phase in task 1. Significant differences (*p* < 0.05) are shown in bold.

EMG (MVC%)	<40	>40	no-BP	cLBP	Age		Pain		Age*Pain	
	Mean ± SD	Mean ± SD	Mean ± SD	Mean ± SD	η²	*P*	η²	*P*	η²	*P*
LMF	39.36 ± 13.6	44.27 ± 14.57	39.96 ± 16.16	43.6 ± 12.78	0.03	0.10	0.02	0.19	0.01	0.25
RMF	37.86 ± 13.82	43.62 ± 11.58	38.47 ± 12.6	42.9 ± 12.84	0.05	**0.02**	0.03	0.08	0.02	0.13
LESL	39.63 ± 18.53	43.62 ± 13	42.6 ± 17.16	41.37 ± 14.76	0.01	0.29	< 0.01	0.66	< 0.01	0.75
RESL	36.63 ± 13.91	41.68 ± 16.48	37.54 ± 15.75	40.79 ± 15.38	0.03	0.11	0.01	0.28	0.01	0.24
LEST	35.74 ± 20.01	41.16 ± 25	37.74 ± 23.19	39.5 ± 23.02	0.02	0.23	< 0.01	0.61	0.02	0.16
REST	37.54 ± 19.27	42.56 ± 32.7	39.33 ± 24.75	41.06 ± 29.61	0.01	0.39	< 0.01	0.65	0.01	0.25
LIO	8.02 ± 7.64	8.18 ± 8.61	7.13 ± 5.01	8.79 ± 9.75	< 0.01	0.76	0.01	0.25	0.03	0.10
RIO	7.6 ± 6.63	11 ± 14.84	7.35 ± 5.63	11 ± 14.79	0.01	0.25	0.02	0.15	< 0.01	0.96
LEO	6.09 ± 3.95	7.4 ± 5.13	6.18 ± 4.08	7.27 ± 5.03	0.02	0.15	0.01	0.25	0.01	0.29
REO	9.19 ± 16.83	8.7 ± 6.44	9.95 ± 17.4	8.2 ± 6.28	< 0.01	0.79	0.01	0.43	0.01	0.34
LRA	5.61 ± 4.45	7.47 ± 8.76	5.94 ± 6.35	7.14 ± 7.78	0.02	0.21	0.01	0.40	0.01	0.38
RRA	5.19 ± 4.16	5.9 ± 4.62	5.35 ± 4.87	5.76 ± 4.11	0.01	0.33	0.02	0.18	0.03	0.10

EMG: electromyography, MVC: maximal voluntary contraction, L/RMF: left/right multifidus, L/RESL: left/right lumbar erector spinae, L/REST: left/right thoracic erector spinae, L/REO: left/right external oblique, L/RIO: left/right internal oblique, and L/RRA: left/right rectus abdominis. η²: Partial eta squared. Significant differences (*p* < 0.05) are shown in bold. Covariates were adjusted as follows: for the effect of age, pain status, sex, and BMI were controlled; for the effect of pain status, age, sex, and BMI were controlled; and for the age-pain status interaction, sex and BMI were controlled.

## Onset time and flexion angles

We further analyzed the effects of age and pain on the onset time and flexion angles for the lifting task. Our findings indicated that none of the onset time and flexion angles demonstrated statistical significance (all *p* > 0.05) (Supplementary Table S1).

### Task2 results

None of the onset times or flexion angles showed statistical significance (Supplementary Table S2). Across the three phases of the lifting task, when analyzing the effect of pain alone and controlling for age, no significant differences were found in EMG amplitudes. Similarly, the combined effects of age and pain were not statistically significant. However, significant differences emerged when focusing on the effect of age while controlling for pain: RMF (*p* = 0.03, η² = 0.05 during the lifting phase), LEST (*p* = 0.03, η² = 0.05 during the lifting phase), REO (*p* = 0.03, η² = 0.05 during the holding phase), and RMF (*p* < 0.01, η² = 0.09 during the lowering phase) (Supplementary Tables S3-5).

### Differences between tasks 1 and 2

Task 1 resulted in higher EMG amplitudes in all back muscles across both pain (no-BP and cLBP) and age (< 40 and > 40) groups compared to Task 2, with significant differences observed during the lifting and holding phases (*p* < 0.05) (Tables 5 and 6). During the lifting phase, ESL muscle activity increased by up to 36% in the no-BP group and 27% in the cLBP group, while participants under 40 showed up to a 28% increase, and those over 40, up to 32%. In the holding phase, ESL activity rose by as much as 1.7 times in the no-BP group and 2 times in the cLBP group, with increases of 1.9 times and 1.8 times for participants under and over 40, respectively. For the lowering phase, EST muscle activity increased by up to 32% in the no-BP group and 25% in the cLBP group, with participants under 40 showing up to a 26% increase and those over 40 showing a 24% rise.

**Table 5:** The differences in EMG amplitude between two lifting tasks by matching pain.

EMG (MVC%)	no-BP		η²	*P*	cLBP		η²	*P*
	Task1	Task2			Task1	Task2		
Lifting								
LMF	57.77 ± 23.62	46.24 ± 18.31	0.06	**0.03**	55.78 ± 15.67	46.26 ± 15.87	0.09	**< 0.01**
RMF	53.59 ± 19.37	44.89 ± 18.99	0.04	0.08	54.91 ± 16.11	46.98 ± 16.71	0.06	**0.01**
LESL	57.25 ± 19.95	43.76 ± 15.68	0.11	**< 0.01**	56.51 ± 18.3	44.47 ± 16	0.12	**< 0.01**
RESL	55.67 ± 26.24	40.64 ± 15.99	0.10	**< 0.01**	54.98 ± 17.55	43.74 ± 16.96	0.10	**< 0.01**
LEST	45.47 ± 25.02	37.94 ± 22.9	0.02	0.26	52 ± 30.55	44.24 ± 21.91	0.02	0.15
REST	47.26 ± 26	40.49 ± 20.32	0.01	0.32	53.75 ± 32.22	45.73 ± 25.46	0.02	0.16
LIO	10.85 ± 12.33	14.76 ± 14.86	0.02	0.20	10.04 ± 11.71	12.08 ± 11.84	0.01	0.27
RIO	8.92 ± 7.43	12.53 ± 9.87	0.04	0.08	11.96 ± 17.93	14.2 ± 16.47	0.01	0.42
LEO	8 ± 5.66	10.37 ± 8.52	0.03	0.10	9.1 ± 7.31	11.96 ± 12.09	0.02	0.13
REO	10.49 ± 9.86	13.9 ± 17.12	0.02	0.23	9.67 ± 8.77	11.55 ± 11.3	0.01	0.31
LRA	6.44 ± 6.02	13.71 ± 12.98	0.09	**0.01**	7.18 ± 5.88	12.95 ± 14.89	0.06	**0.01**
RRA	7.31 ± 9.65	19.5 ± 37.51	0.05	0.05	6.69 ± 5.11	12.83 ± 16.33	0.06	**0.01**
Holding								
LMF	31.74 ± 14.11	14.06 ± 11.89	0.32	**< 0.01**	30.61 ± 10.18	12.57 ± 8.17	0.47	**< 0.01**
RMF	28.84 ± 11.83	12.16 ± 10.31	0.37	**< 0.01**	29.51 ± 9.77	12.54 ± 9.44	0.43	**< 0.01**
LESL	30.43 ± 18.4	11.27 ± 10.4	0.30	**< 0.01**	27.52 ± 11.45	9.06 ± 7.92	0.47	**< 0.01**
RESL	25.3 ± 13.91	10.72 ± 9.52	0.28	**< 0.01**	25.1 ± 10.98	9.62 ± 8.44	0.38	**< 0.01**
LEST	29.49 ± 15.83	12.47 ± 10.19	0.28	**< 0.01**	31.57 ± 17.97	14.48 ± 14.54	0.21	**< 0.01**
REST	30.59 ± 16.21	14.85 ± 10.6	0.24	**< 0.01**	29.08 ± 17.95	13.21 ± 12.14	0.21	**< 0.01**
LIO	7.74 ± 6.56	6.47 ± 4.48	0.01	0.29	9.27 ± 11.83	7.22 ± 7.93	< 0.01	0.45
RIO	7.41 ± 7.5	6.11 ± 4.34	0.01	0.36	10.77 ± 15.69	8.86 ± 14.85	< 0.01	0.66
LEO	5.67 ± 4.59	5.88 ± 4.08	< 0.01	0.76	7.55 ± 7.32	6.53 ± 4.82	0.01	0.44
REO	6.82 ± 5.12	6.54 ± 4.03	< 0.01	0.80	7.8 ± 8.3	6.61 ± 4.84	0.01	0.42
LRA	4.1 ± 4.57	4.53 ± 5.11	< 0.01	0.60	3.93 ± 3.03	3.44 ± 2.26	< 0.01	0.84
RRA	3.71 ± 4.63	3.74 ± 3.81	< 0.01	0.89	4.13 ± 3.26	3.61 ± 2.71	< 0.01	0.64
Lowering								
LMF	39.96 ± 16.16	38.21 ± 13.9	< 0.01	0.66	43.6 ± 12.78	40.34 ± 13.75	0.02	0.14
RMF	38.47 ± 12.6	38.28 ± 16.67	< 0.01	0.99	42.9 ± 12.84	40.35 ± 14.28	0.01	0.27
LESL	42.6 ± 17.16	36.7 ± 13.23	0.03	0.09	41.37 ± 14.76	37.92 ± 13.51	0.02	0.16
RESL	37.54 ± 15.75	34.92 ± 15.51	0.01	0.48	40.79 ± 15.38	36.43 ± 14.85	0.02	0.12
LEST	37.74 ± 23.19	28.59 ± 15.62	0.04	0.06	39.5 ± 23.02	35.1 ± 19.8	0.01	0.37
REST	39.33 ± 24.75	32.33 ± 18.3	0.02	0.20	41.06 ± 29.61	32.93 ± 18.13	0.02	0.10
LIO	7.13 ± 5.01	12.95 ± 10.62	0.11	**< 0.01**	8.79 ± 9.75	10.81 ± 9.8	0.01	0.21
RIO	7.35 ± 5.63	11.8 ± 9.76	0.07	**0.01**	11 ± 14.79	14.38 ± 17.44	0.01	0.25
LEO	6.18 ± 4.08	10.5 ± 6.68	0.14	**< 0.01**	7.27 ± 5.03	11.37 ± 9.41	0.07	**< 0.01**
REO	9.95 ± 17.4	10.95 ± 6.93	< 0.01	0.69	8.2 ± 6.28	11.06 ± 8.39	0.03	**0.04**
LRA	5.94 ± 6.35	12.96 ± 13.42	0.09	**< 0.01**	7.14 ± 7.78	11.33 ± 13.75	0.04	**0.03**
RRA	5.35 ± 4.87	25.27 ± 63.43	0.05	0.05	5.76 ± 4.11	11.49 ± 12.79	0.09	**< 0.01**

EMG: electromyography, MVC: maximal voluntary contraction, L/RMF: left/right multifidus, L/RESL: left/right lumbar erector spinae, L/REST: left/right thoracic erector spinae, L/REO: left/right external oblique, L/RIO: left/right internal oblique, and L/RRA: left/right rectus abdominis. η²: Partial eta squared. Significant differences (*p* < 0.05) are shown in bold.

**Table 6:** The differences in EMG amplitude between two lifting tasks by matching age.

EMG (MVC%)	< 40		η²	*P*	> 40		η²	*P*
	Task1	Task2			Task1	Task2		
Lifting								
LMF	53.04 ± 15.84	43.06 ± 11.51	0.10	**< 0.01**	59.37 ± 21.23	48.75 ± 19.83	0.06	**0.01**
RMF	50.98 ± 19.9	41.91 ± 15.24	0.06	**0.02**	57.02 ± 14.88	49.35 ± 18.83	0.04	**0.02**
LESL	52.12 ± 22	42.03 ± 14.73	0.07	**0.01**	60.47 ± 15.28	45.83 ± 16.5	0.17	**< 0.01**
RESL	51.61 ± 21.36	40.06 ± 16.05	0.09	**0.01**	58.11 ± 21.19	44.25 ± 16.82	0.11	**< 0.01**
LEST	43.23 ± 24.37	35.79 ± 16.95	0.02	0.15	54.1 ± 30.68	46.04 ± 25.19	0.01	0.21
REST	47.33 ± 27.18	41.34 ± 22.5	0.01	0.32	54.04 ± 31.74	45.16 ± 24.21	0.02	0.16
LIO	9.19 ± 7.29	13.79 ± 11.67	0.06	**0.02**	11.3 ± 14.54	12.8 ± 14.39	< 0.01	0.57
RIO	8.36 ± 5.85	13.98 ± 12.25	0.09	**< 0.01**	12.56 ± 18.67	13.1 ± 15.31	< 0.01	0.88
LEO	7.16 ± 4.91	11.27 ± 11.45	0.06	**0.03**	9.81 ± 7.63	11.28 ± 10.16	0.01	0.35
REO	8.97 ± 9.17	14.27 ± 17.63	0.04	0.07	10.82 ± 9.21	11.21 ± 10.44	< 0.01	0.81
LRA	5.88 ± 4.08	14.1 ± 14.45	0.14	**< 0.01**	7.66 ± 6.96	12.63 ± 13.81	0.04	**0.03**
RRA	6.71 ± 9.13	20.54 ± 38.6	0.06	**0.02**	7.13 ± 5.48	11.89 ± 12.93	0.04	**0.03**
Holding								
LMF	27 ± 10.07	11.67 ± 7.72	0.41	**< 0.01**	34.26 ± 12.3	14.41 ± 11.26	0.41	**< 0.01**
RMF	26.11 ± 11.04	10.85 ± 7.73	0.39	**< 0.01**	31.68 ± 9.67	13.58 ± 11.03	0.43	**< 0.01**
LESL	26.1 ± 17.6	8.83 ± 7.27	0.29	**< 0.01**	30.75 ± 11.65	10.92 ± 10.25	0.45	**< 0.01**
RESL	21.94 ± 11.45	9.16 ± 8.11	0.29	**< 0.01**	27.71 ± 12.25	10.82 ± 9.47	0.37	**< 0.01**
LEST	26.76 ± 16.03	10.95 ± 9.91	0.26	**< 0.01**	33.81 ± 17.37	15.7 ± 14.47	0.23	**< 0.01**
REST	27.36 ± 14.91	12.13 ± 9.28	0.27	**< 0.01**	31.52 ± 18.72	15.31 ± 12.85	0.20	**< 0.01**
LIO	8.46 ± 7.4	6.76 ± 5.3	0.01	0.38	8.79 ± 11.71	7.01 ± 7.59	0.01	0.43
RIO	7.2 ± 5.84	6 ± 3.38	< 0.01	0.77	11.12 ± 16.49	8.99 ± 15.15	< 0.01	0.52
LEO	5.33 ± 4.41	5.34 ± 3.63	< 0.01	0.76	7.92 ± 7.43	6.96 ± 5	0.01	0.44
REO	5.58 ± 3.92	5.44 ± 3.11	< 0.01	0.82	8.83 ± 8.68	7.47 ± 5.18	0.01	0.31
LRA	3.37 ± 3.27	3.57 ± 2.94	0.01	0.42	4.49 ± 3.98	4.17 ± 4.32	< 0.01	0.82
RRA	3.16 ± 2.47	3.43 ± 2.71	0.01	0.38	4.58 ± 4.6	3.85 ± 3.57	< 0.01	0.45
Lowering								
LMF	39.36 ± 13.6	36.53 ± 10.1	0.01	0.31	44.27 ± 14.57	41.69 ± 15.82	0.01	0.33
RMF	37.86 ± 13.82	34.5 ± 12.7	0.01	0.27	43.62 ± 11.58	43.34 ± 16.12	< 0.01	0.88
LESL	39.63 ± 18.53	34.49 ± 12.55	0.03	0.11	43.62 ± 13	39.67 ± 13.59	0.02	0.15
RESL	36.63 ± 13.91	32.95 ± 13.69	0.02	0.20	41.68 ± 16.48	37.99 ± 15.84	0.01	0.28
LEST	35.74 ± 20.01	28.42 ± 15.37	0.03	0.09	41.16 ± 25	35.36 ± 19.96	0.01	0.27
REST	37.54 ± 19.27	30.41 ± 17.86	0.03	0.13	42.56 ± 32.7	34.44 ± 18.27	0.02	0.13
LIO	8.02 ± 7.64	13.35 ± 11.97	0.07	**0.01**	8.18 ± 8.61	10.46 ± 8.38	0.02	0.15
RIO	7.6 ± 6.63	13.12 ± 12.56	0.08	**0.01**	11 ± 14.84	13.4 ± 16.2	0.01	0.42
LEO	6.09 ± 3.95	12.05 ± 9.25	0.16	**< 0.01**	7.4 ± 5.13	10.17 ± 7.5	0.04	0.03
REO	9.19 ± 16.83	11.86 ± 8.8	0.01	0.33	8.7 ± 6.44	10.35 ± 6.86	0.01	0.22
LRA	5.61 ± 4.45	13.31 ± 14.92	0.12	**< 0.01**	7.47 ± 8.76	11.02 ± 12.46	0.02	0.10
RRA	5.19 ± 4.16	22.26 ± 58.82	0.04	0.06	5.9 ± 4.62	13.59 ± 24.11	0.04	**0.03**

EMG: electromyography, MVC: maximal voluntary contraction, L/RMF: left/right multifidus, L/RESL: left/right lumbar erector spinae, L/REST: left/right thoracic erector spinae, L/REO: left/right external oblique, L/RIO: left/right internal oblique, and L/RRA: left/right rectus abdominis. η²: Partial eta squared. Significant differences (*p* < 0.05) are shown in bold.

In contrast to the back muscles, the activation patterns of the abdominal muscles were less consistent. For some muscles, Task 2 demonstrated significantly higher activation during lifting and lowering phases (*P* < 0.05) across both pain (no-BP and cLBP) and age (< 40 and > 40) groups (Tables 5 and 6). Specifically, during the lifting phase, RA activity increased by up to 1.6 times and 92% in the no-BP and cLBP groups, respectively, and by up to 2 times and 67% in participants under and over 40 years of age, respectively. In the lowering phase, ESL activity increased as much as 3.7 times in the no-BP group and 99% in the cLBP group, with a rise of 3.3 times and 1.3 times in participants under and over 40, respectively.

## Discussion

This study aimed to investigate the effect of age and cLBP on EMG patterns and flexion angles during various lifting tasks. Our findings underscore the significant influence of age on muscle activation, with distinct patterns emerging across different tasks. Furthermore, almost no significant differences were observed in the EMG activities and flexion angles between the no-BP and cLBP groups.

When we controlled for age, sex, and BMI to examine the effect of pain on EMG, we found no significant differences in muscle activation between the cLBP group and those without cLBP. This contrasts with previous studies^17,24,25^which have reported significantly higher EMG amplitudes in individuals with cLBP. One possible explanation for the increased EMG activity observed in prior research is muscle weakness, as documented by Cassisi et al.^40^who reported reduced strength in individuals with cLBP. Due to this back muscle weakness, individuals in the cLBP group required greater activation of the back muscles to maintain equilibrium and an upright trunk posture against the trunk flexion moment. Additionally, the overall increase in activation of both back and abdominal muscles may improve lumbopelvic stability, this co-contraction of back and abdominal muscles, observed in cLBP individuals^41,42^has been suggested as a motor control strategy for stabilizing the lumbopelvic region^42^. This explanation may also account for why, although our findings did not reach statistical significance, EMG amplitudes tended to be higher in most muscles in the cLBP group compared to the no-BP group, particularly in the abdominal muscles and some back muscles during the lowering phase.

Furthermore, in contrast to previous study^7^we did not observe significant differences in MF activation between the two groups. Some studies, including Wesselink et al.^43^have highlighted that the MF at L5 is particularly susceptible to degenerative structural and functional changes in cLBP. However, our results did not reveal EMG differences at this segment. Moreover, the study on the association between EMG activity and paraspinal muscle degeneration found no significant association between MF activation and muscle degeneration at L5^44^. The absence of differences in MF activation in our study may reflect the heterogeneity of cLBP populations, methodological differences across studies, or compensatory mechanisms, such as increased co-contraction of other trunk muscles, which could obscure changes in MF activity. Additionally, the relatively mild to moderate pain intensity in our cLBP participants may have contributed to the lack of observed differences. Further research is needed to clarify the factors underlying these inconsistencies.

However, our study found that the cLBP group exhibited a significantly slower lifting velocity compared to the no-BP group. This finding is consistent with the results of other studies^25,45^which may be attributed to the weakness of the trunk extensor muscles^46,47^ and gluteus maximus^48,49^ in patients with cLBP. Additionally, cLBP patients experience anxiety and fear related to their pain (the cLBP group showed significantly higher values in TSK and FABQ-PA scores). Moreover, participants with higher RMDQ scores (the cLBP group showed significantly higher values in RMDQ scores) demonstrated greater global stiffness of the lumbar spine^50^. Consequently, patients with cLBP require more time to complete tasks involving lifting or moving objects.

In addition, we excluded confounding factors such as pain, sex, and BMI and analyzed the effect of age on EMG. The results demonstrated that age significantly affects muscle activation, particularly during the holding phase of lifting tasks. Specifically, younger participants exhibited lower EMG amplitudes compared to older participants in several muscles, LMF, RMF, LEST, RESL, LEO, REO, and LRA. Notably, our study indicates no statistically significant differences when analyzing the effect of pain alone or the combined effects of age and pain; thus, our results suggest that age is an independent factor affecting EMG amplitude. Our study aligns with the findings of an earlier study^29^which reported significant differences in amplitudes recorded between the standing and maximum flexion positions across age groups, with older patients showing higher values compared to the younger group. These findings may be attributed to age-related changes in muscle mass, composition, contractile properties, and tendon function^26^. Such changes lead to reduced muscle power and strength^26^. For instance, a decline in tendon stiffness with age can lower the rate of force production during muscle contraction^13,26^. Consequently, older adults require greater muscle activation to partially compensate for these changes, thereby maintaining postural stability and motor function.

Additionally, in a study on anticipatory postural control in older adults^51^it was found that the activation of the ESL occurred later in older patients compared to younger individuals. This delay may be due to age-related impairments in neural structures responsible for detecting postural instability, such as the supplementary motor area and the foot area of the sensorimotor cortex^52^. In addition, the age-related decline in muscle mass is due to the loss of both slow and fast motor units, with a more rapid reduction in fast motor units^26,53^. These impairments likely contribute to increased postural instability in older adults, requiring them more time to prepare for movement. However, our study did not find significant differences in the onset time of EMG. The absence of significance may be due to the age range of our participants, as we only included patients aged 19–64, with the average age of our older group being 51.38 years.

The comparison in EMG amplitudes between the two tasks showed that Task 1 had significantly higher activation in most back muscles, with back muscle activity increasing by up to 2 times compared to Task 2. This pattern was observed across both pain groups (no-BP and cLBP) and age groups (< 40 and > 40), particularly during the lifting and holding phases (*p* < 0.05). This difference may be attributed to the following reason: Task 1 involved lifting a 10 kg box from the ground to hip height, which generated a significant moment arm due to the anterior position of the load relative to the body’s center of gravity. Task 2, on the other hand, involved lifting two 5 kg dumbbells from the ground and holding them at the sides of the body, where the moment arms are shorter, reducing the mechanical demand on the back muscles. Although the total load is the same (10 kg), its distribution between both hands in Task 2 minimizes the moment arm^8^ and, consequently, the load on individual muscle groups, particularly the back muscles^9^. The observation is supported by previous studies that show muscle activity increases with rising loads^12^. When the load is held in front of the body with both hands, the center of mass of the upper body shifts anteriorly^9^. This shift creates a significantly increased moment^8^requiring the back muscles to generate greater force^9^ and increased muscle activity^8^ for stabilization and lifting. Furthermore, spine stability depends on the relative activation of all trunk muscles, as no single muscle can be identified as the most crucial for lumbar spine stability^54^. These explained why in Task 1, nearly all back muscles exhibit significantly higher EMG amplitudes compared to Task 2. In contrast, Task 2 showed greater abdominal muscle contraction during both the lifting and lowering phases, with EMG in abdominal muscle increasing by up to 3.7 times compared to Task 1, showing significant differences across both pain and age groups. The varying activation levels of back and abdominal muscles across different tasks and phases suggest that interventions should be tailored to meet the specific demands on these muscle groups during various activities.

While our study provided important findings, several limitations should be noted. First, the cLBP group had relatively mild to moderate pain intensity, which may not cover the full range of cLBP severity. Second, aspects such as the duration of cLBP and occupational influences, which might affect EMG and flexion angles, were not thoroughly investigated. Additionally, surface EMG recordings were susceptible to cross-talk artifacts. Future research with larger and more diverse groups and comprehensive evaluations could offer more detailed insights.

## Conclusions

The significant differences in EMG amplitudes observed in older participants suggest that age-related factors may contribute to altered neuromuscular responses, which could influence injury risk and rehabilitation outcomes. Additionally, The absence of significant differences in EMG between the cLBP and no-back pain groups, after controlling for confounding variables, highlights the complexity of pain perception and muscle activation in individuals with cLBP. Furthermore, the variation in EMG patterns between different lifting tasks emphasizes the importance of task specificity in both assessment and treatment planning. Given that some of the observed neuromuscular adaptations may be linked to muscle weakness, it is essential to integrate age-appropriate strengthening programs targeting both deep and superficial trunk muscles into treatment protocols. Such age and pain related interventions may help improve lumbopelvic stability, mitigate functional impairments, and enhance long-term clinical outcomes in individuals with cLBP. Additional investigation is required to get deeper into basic mechanisms and devise specific therapies for various age cohorts.

## Data Availability

The data presented in this study are available upon reasonable request from the corresponding author.
